# Are treatment effects of neurofeedback training in children with ADHD related to the successful regulation of brain activity? A review on the learning of regulation of brain activity and a contribution to the discussion on specificity

**DOI:** 10.3389/fnhum.2015.00135

**Published:** 2015-03-27

**Authors:** Agnieszka Zuberer, Daniel Brandeis, Renate Drechsler

**Affiliations:** ^1^Department of Child and Adolescent Psychiatry, University of ZurichZurich, Switzerland; ^2^Neuroscience Center Zurich, University of Zurich and ETH ZurichZurich, Switzerland; ^3^Department of Child and Adolescent Psychiatry and Psychotherapy, Central Institute of Mental Health, Medical Faculty Mannheim/ Heidelberg UniversityMannheim, Germany; ^4^Center for Integrative Human Physiology, University of ZurichZurich, Switzerland

**Keywords:** neurofeedback, ADHD, specificity, self-regulated brain activity, learning curves, learning indices

## Abstract

While issues of efficacy and specificity are crucial for the future of neurofeedback training, there may be alternative designs and control analyses to circumvent the methodological and ethical problems associated with double-blind placebo studies. Surprisingly, most NF studies do not report the most immediate result of their NF training, i.e., whether or not children with ADHD gain control over their brain activity during the training sessions. For the investigation of specificity, however, it seems essential to analyze the learning and adaptation processes that take place in the course of the training and to relate improvements in self-regulated brain activity across training sessions to behavioral, neuropsychological and electrophysiological outcomes. To this aim, a review of studies on neurofeedback training with ADHD patients which include the analysis of learning across training sessions or relate training performance to outcome is presented. Methods on how to evaluate and quantify learning of EEG regulation over time are discussed. “Non-learning” has been reported in a small number of ADHD-studies, but has not been a focus of general methodological discussion so far. For this reason, selected results from the brain-computer interface (BCI) research on the so-called “brain-computer illiteracy”, the inability to gain control over one’s brain activity, are also included. It is concluded that in the discussion on specificity, more attention should be devoted to the analysis of EEG regulation performance in the course of the training and its impact on clinical outcome. It is necessary to improve the knowledge on characteristic cross-session and within-session learning trajectories in ADHD and to provide the best conditions for learning.

Recent meta-analyses and reviews have evaluated the efficacy of neurofeedback training in children and have concluded that there is a need for more placebo-controlled studies in ADHD research with better blinding of raters and possibly also of trainers (Lofthouse et al., 2012; Sonuga-Barke et al., 2013). Placebo control, often used interchangeably with sham (e.g., Heywood and Beale, 2003; van Dongen-Boomsma et al., 2013) or mock (e.g., Egner et al., 2002) feedback in this context, lacks only the active core component, namely the consistent feedback contingent upon specific EEG patterns, and appears indistinguishable from the neurofeedback condition. This typically implies that non-contingent sham feedback is provided to the participant during the training, either by frequently changing contingencies with real data (e.g., Heywood and Beale, 2003), by using simulated EEG-like data or feedback (e.g., Logemann et al., 2010; van Dongen-Boomsma et al., 2013), or pre-recorded data, which all may be combined with contingent feedback of real artifacts (Kerson and Collaborative Neurofeedback Group, 2013). While placebo control and pre-post analyses of change on clinical, neuropsychological and electrophysiological levels would appear to be the first choice with regard to efficacy, it may be questioned whether they constitute the best method for investigating the specificity of NF. Although placebo control aims to control for all non-specific influences of the training setting, such as learning to sit still, improved personal well-being due to the positive relation to the therapist, or positive expectations, it entails methodological limitations. Sham feedback fails to control for generic and non-specific learning effects, i.e., by the experience of improvement and progressive mastery, of self-efficacy, and increase of control which may be induced by any kind of biofeedback. Although sham neurofeedback using slowly alternating contingencies with different frequencies may allow at least piecewise learning (Hoedlmoser et al., 2008; Doppelmayr et al., 2009), alternative placebo-type control conditions such as EMG biofeedback (Bakhshayesh et al., 2011; Maurizio et al., 2014), or feedback from a distinct control region as in neuroimaging (Caria et al., 2007) provide better control for progressive learning.

More importantly, with regard to specificity, neither placebo control nor any other type of control condition can provide positive proof that successful learning of EEG regulation in the active condition is responsible for clinical improvements. To that aim, it would be necessary to demonstrate that learning of EEG-regulation occurred during the training and that the NF-training success, in the sense of successfully learned self-regulation of brain activity across time, is related to positive outcome on the clinical, neuropsychological or electrophysiological level (see Holtmann et al., 2014a). Adequate control for the generic effects of learning would then require successful learning at a similar rate in the control condition.

In addition, for the time being, the effects which might be induced by sham feedback remain poorly understood. This may be particularly relevant for individuals with ADHD, who according to the ADHD literature may display problems with self-perception in various different ways: A sizable portion of children with ADHD show an inappropriate overestimation of self-efficacy and ability, the so-called illusory positive bias (see Owens et al., 2007). Other studies have demonstrated feelings of low self-efficacy and low self-esteem in patients with ADHD (Newark and Stieglitz, 2010; Mazzone et al., 2013) which usually leads to a negative bias in self-perception. In addition, patients with ADHD seem to display problems with the self-perception of internal states (Donfrancesco et al., 2013). Many children with ADHD may be unaware of how it feels to be in an alert and focussed state of mind. Thus, providing ADHD patients with sham feedback could prevent them from developing a more adequate self-perception or lead them to mistrust their intuition. Although the findings from sham neurofeedback control conditions suggest no detrimental effects regarding core ADHD symptoms, effects on self-perception remain to be tested directly. Also from this perspective, NF studies which use genuine neurofeedback and which examine whether learning of self-regulated EEG activity actually occurred during the training, may present a better alternative in order to investigate the specificity of NF than placebo controlled studies.

In this paper, we will present a short review of NF-studies with ADHD patients in which learning of EEG regulation was analyzed and we will discuss methods how to evaluate and quantify learning of EEG regulation over time. Among the many varieties of NF protocols with ADHD (e.g., Hurt et al., 2014), the training of frequency bands (NF-FB) and the training of slow cortical potentials (NF-SCP) are the best scientifically evaluated and will therefore be the focus of the following review (Table 1). We will additionally refer to studies with Q-EEG-training and with healthy participants or clinical groups other than ADHD in order to illustrate a respective method (Figure 1).

**Table 1:** **ADHD Neurofeedback studies analyzing learning of EEG regulation**.

Study, NF-participants	Protocol, electrode sites, no. of sessions	Learning parameter/criterion for good performance	Learner rates/learning outcome	Association between NF-learning and outcome gains
Lubar et al. (1995) *N* = 17	Theta↓/Beta↑ (bipolar electrodes situated halfway between Cz and Pz and halfway between Fz and Pz); 40 sessions	MP Theta/Beta/significant positive correlation between sessional learning parameter and session number	65% learners	Stronger improvement in attentional test (TOVA) in learners than non-learners
Kropotov et al. (2005) *N* = 86	Beta↑ (C3-Fz); SMR↑ (C4-Pz); 15–22 sessions	At least 25 % increase of within sessional Beta- or SMR-power relative to resting-BL at the 1st session/of at least 60 % of successful sessions	82.5% learners	Improvements of ADHD symptoms and of Go/Nogo response-time and Go/Nogo SD
Strehl et al. (2006) *N* = 25 (Gani et al., 2008: 2-years-follow up)	SCP ↑↓ (Cz); 30 sessions (3 blocks of 10); follow-up sessions 31–33 (after 6 months)	MA of negativity trials during FB and TF, difference in MA between positivity and negativity trials/Good and poor learners based on median split of mean difference between MA of positivity and negativity trials at 3rd training phase	*MA negativity trials*: 2nd session < last session 2nd session < follow-up *Difference between MA of positivity and negativity trials*: at follow-up↑	Good TF-performance (difference between MA of positive and negative trials, sessions 21–30) is associated with clinical improvement only in good learners
Drechsler et al. (2007) *N* = 17 (Doehnert et al., 2008)	SCP ↑↓ (Cz); 30 sessions	MA of negativity trials during FB and TF/good and poor learners based on median split of mean difference between MA of positivity and negativity trials during TF-sessions 14–28	*MA negativity trials*: FB: session 3–6 < session 25–28 TF: session 3–6 < session 25–28	Difference between MA of positive and negative trials during TF (sessions 14–28) correlates with clinical improvements (hyperactivity/impulsivity) in good learners
Leins et al. (2007) Group 1 *N* = 16, Group 2 *N* = 16	Group 1: Theta↓(↑), Beta↑(↓) (C3f, C4f); Group 2: SCP↑↓ (Cz); 30 sessions, 31–33 follow-up sessions (after 6 months)	Group 1: MP Theta/Beta Group 2: MA of negativity trials. Both: difference between up- und down-regulation	EEG learning both groups: 2nd session < last session 2nd session < follow up
Bakhshayesh et al. (2011) *N* = 18	Theta↓/Beta↑ (Cz); session BL; 30 sessions	MP Theta/Beta across sessions (section 1, 2, 3)	Theta/Beta ↓ in 2 out of 3 training conditions; BL ↓	
DeBeus and Kaiser (2011) *N* = 42	Beta↑/(Theta + Alpha)↓ (Fz); 20 sessions	[Beta/(Theta + Alpha)] ↑ ( = Engagement Index) of sessions 1–3 compared to 18–20/Increase of Engagement Index by $\frac{1}{2}$ SD	74% learners	Teacher rated improvements correlate with change in Engagement Index in learners
Liechti et al. (2012) Maurizio et al. (2014) *N* = 13	Theta↓(↑)/Beta↑(↓); SCP↑↓; tomographic NF of anterior cingulate cortex activity; Pre-session QEEG ; 36 sessions	MP of Beta/Theta or MA across sessions	Only partial learning for a simple SCP variant, otherwise no cross-sessional learning; decrease of pre-session QEEG within-NF-group variability across sessions (normalization)	No association between EEG learning and behavioral outcome, except between SCP delayed feedback regulation and hyperactivity/impulsivity
Hillard et al. (2013) *N* = 18	Undisclosed protocol (wide band spectrum regulation) (Fpz); 12 sessions	MP frequency analysis at FPz within (minute 1 to 25) and across sessions (session 1 to 12)	*Across sessions*: Alpha↑ and Beta↓, all other frequency bands↓; *Within session*: Theta/Beta ↓, Theta/Alpha ↓
Russell-Chapin et al. (2013) *N* = 12	SMR↑ (Cz); 40 sessions	MP of SMR	SMR↑ (session 1 < session 40)	
Bink et al. (2014) *N* = 45 (adolescents)	Theta↓/SMR↑ (Cz); Session mean 37 (± 5)	MP of Theta/SMR (Alpha, high Beta) of sessions 1–5 compared to 31–35; Within session first 15 min. compared to last 15 min.	*Across session*: no change of overall MP; *Within-session*: Theta↓ larger at sessions 31–35 than 1–5.
Escolano et al. (2014) *N* = 20	Individual upper Alpha↑ (AFz, F3, Fz, F4, FCz and Cz); Pre- and post-session active and passive BL; 18 sessions	MP of individual upper Alpha across sessions and within sessions	Pre-session task-related MP ↑ (= active BL) across sessions; Pre-post MP ↓ within sessions; absolute and relative Alpha MP ↓ within sessions	No association between learning/training response and behavioral improvements
Gevensleben et al. (2014) *N* = 10	SCP ↑↓ (Cz); 13 double sessions	MA during positivity or negativity trials/MA↑ across sessions 1, 5, 9 and 13	Cross sessional increase of negative MA during negativity trials	Association between negativity MA of session 5 and 9 and inattention symptoms↓
Takahashi et al. (2014) *N* = 10	SCP ↑↓ (Cz); 16 (20) sessions	Peak amplitude during positivity or negativity trials across sessions	Positive shift amplitude ↑ in session 9, 13; negative shift amplitude ↑ in session 11, 12
Vollebregt et al. (2014) EEG learning analyzed: *N* = 10	Individualized protocols; most often SMR↑/Theta↓; 30 sessions	MP per trained frequency-band across sessions	No systematic improvement on target frequencies

*SCP = slow cortical potentials, MA = mean amplitude, MP = mean power, ↓ = decrease, ↑ = increase, TF = transfer condition, FB = feedback condition, BL = baseline, SD = standard deviation*.

**Figure 1:**
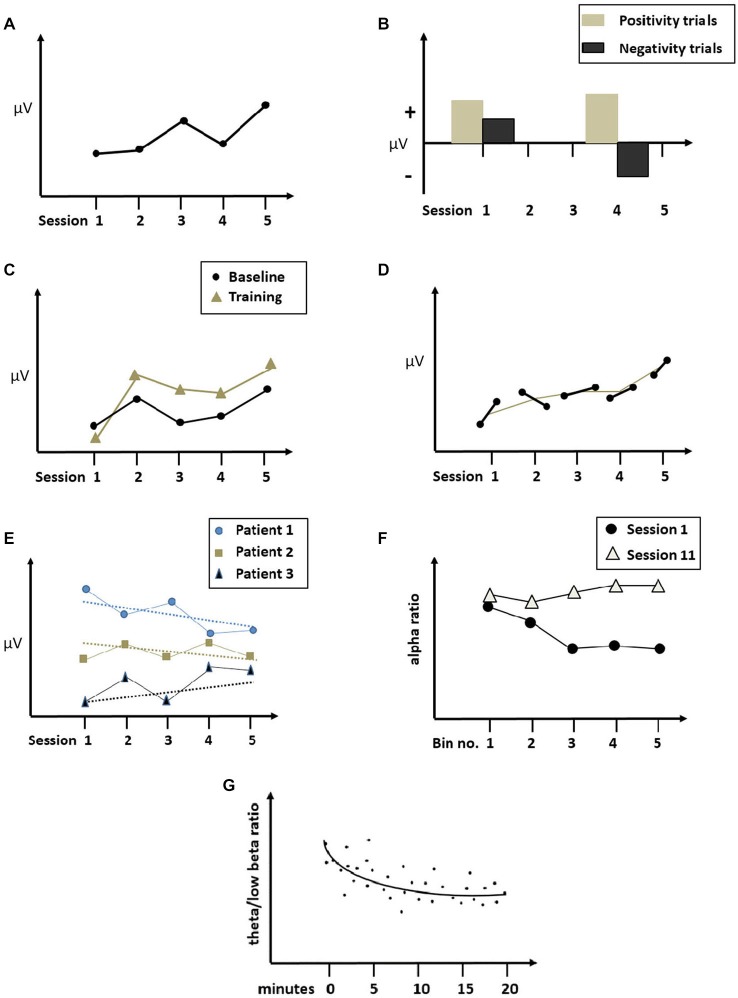
**Illustration of across and within session learning curves**. **(A)** Across sessions comparison of single sessions (SCP mean amplitude during positivity and negativity trials; adapted from Gevensleben et al., 2014; modified). **(B)** Learning curve across sessions of mean training performance (e.g., Cho et al., 2008; modified). **(C)** Pre-session baseline and mean training performance across sessions (adapted from Dempster and Vernon, 2009, modified), **(D)** Pre- and post- session baselines across sessions (adapted from Escolano et al., 2011, modified). **(E)** Individual pre-session baselines across sessions (adapted from Liechti et al., 2012, modified). **(F)** Within session learning curves of training performance during session 1 and session 11, segmented into bins of time (adapted from Cho et al., 2008, modified). **(G)** Within session learning curve collapsed across sessions, indicating mean theta/beta ratio per minute (adapted from Hillard et al., 2013, modified). The figures illustrate the methods used in the studies; all data have been modified.

## ADHD Neurofeedback Protocols and Learning of EEG Self-Regulation

We identified 15 published NF group studies with ADHD children which include the analysis of EEG regulation learning across training sessions (Table 1). The majority of these studies used NF training of the frequency bands (NF-FR) and central electrodes. During NF-FR, subjects are provided with continuous (visual or/and audio) feedback and are positively reinforced as long as the spectral activity of the targeted EEG-frequency band or the ratio of specific frequency bands stays below (or above, respectively) a pre-defined threshold. As soon as the threshold is passed, the feedback stimulus changes, announcing that the subject has reached an undesired state. A classic ADHD study protocol aims to decrease theta activity and increase beta activity (Lubar et al., 1995; Leins et al., 2007; Bakhshayesh et al., 2011). Another characteristic protocol for ADHD aims at increasing the sensorimotor rhythm (SMR; Kropotov et al., 2005; Russell-Chapin et al., 2013), which is known to play an important role for motor excitability (Sterman et al., 1970; Pfurtscheller et al., 1996). While these frequency specific protocols are usually employed with the aim of obtaining “normalization” of characteristic spectral EEG abnormalities in ADHD, a more recent rationale is to train “regulation” of spectral EEG activity instead (Holtmann et al., 2009, 2014a). This change in perspective is based on research that failed to find consistently abnormal or characteristic EEG frequency patterns in children with ADHD at group level (Liechti et al., 2013). Consequently, some NF-FR protocols alternate between phases of up- and down-regulation which is consistent with the typical approach in SCP regulation (Leins et al., 2007; Liechti et al., 2012; Maurizio et al., 2014). In contrast, QEEG NF training (and/or z-score training) and other individualized NF protocols assume EEG abnormalities compared to normative data, which are trained in order to reach normalization (Hillard et al., 2013; Vollebregt et al., 2014).

In six out of 15 studies, NF of the slow cortical potentials (NF-SCP) was used (Strehl et al., 2006; Drechsler et al., 2007; Gevensleben et al., 2014; Takahashi et al., 2014), sometimes in combination (Liechti et al., 2012; Maurizio et al., 2014) or contrasted with NF-FR (Leins et al., 2007). SCPs are shifts in electro-cortical potentials which are thought to index the regulation of cortical excitability. NF-SCP trials are short, at about 8 s, and participants are instructed to enhance activation (negativity trials) or reduce activation (positivity trials) relative to the baseline measured at the beginning of each trial. The magnitude of a produced negative amplitude reflects the amount of resources allocated to prepare a motor or cognitive response while a shift towards the positive polarity reflects a decrease in cortical excitability, which is in turn associated with a reduced responsiveness (Birbaumer et al., 1990).

In these NF-SCP studies, learning progress was mostly confined to negativity trials (i.e., to activation), while no or only moderate learning seemed to occur in positivity trials (i.e., deactivation) (Strehl et al., 2006; Drechsler et al., 2007; Leins et al., 2007; Gevensleben et al., 2014; for NF-SCP with healthy adults see Studer et al., 2014). In the initial training sessions, subjects seemed to spontaneously produce positive amplitudes (Strehl et al., 2006; Drechsler et al., 2007), but failed to do so in the subsequent sessions, possibly because they took recourse to more intentional strategies. According to Strehl et al. (2006), children report that the positivity trials are more difficult and exhausting. Alternatively, considering the already high performance in positivity trials during the initial training phase, the lack of improvement in positivity trials might be attributed to a possible ceiling effect (Strehl et al., 2006; Leins et al., 2007). Only one recent study (Takahashi et al., 2014) found comparable increase of positive as well as negative shift amplitudes across training, based on peak amplitudes.

Very few ADHD-studies examined learning of EEG regulation in transfer conditions (Strehl et al., 2006; Drechsler et al., 2007; Leins et al., 2007; Liechti et al., 2012) which is hypothesized to be a more ecologically valid learning measure than performance in feedback trials. In transfer trials, participants regulate their brain activity without feedback or while feedback is delayed. The ability to follow the instructions during transfer trials without the aid of immediate feedback should reflect the child’s ability to regulate his/her brain activity independently of external triggers. This ability is considered a necessary precondition for applying the acquired skill in situations outside the laboratory. NF-studies in ADHD reporting learning progress for both transfer and feedback trials are rare (Leins et al., 2007; Table 1). There is evidence that ADHD-subjects are less effective in transfer trials than in feedback trials (Strehl et al., 2006; Drechsler et al., 2007; Leins et al., 2007), which also appears to be the case in patients suffering from epilepsy (Kotchoubey et al., 1999). In healthy adults, EEG regulation performances were also less effective during transfer (Rockstroh et al., 1990) or comparable in both types of trials (Lutzenberger et al., 1982).

Several ADHD-studies compared NF-learning to learning progress in other biofeedback modalities, such as muscle relaxation (Bakhshayesh et al., 2011; for a recently published study design see Holtmann et al., 2014b) or biofeedback-guided learning of fine motor skills (Maurizio et al., 2014), with the latter showing better learning with motor than with EEG feedback. Liechti et al. (2012) reported that children with ADHD did not display learning of EEG regulation across sessions in a tomographic EEG NF training. However, they did show progressive learning in muscular artifact control, thus demonstrating a significantly improved ability to sit still.

## Measuring Learning of EEG Self-Regulation

As indicated in Table 1 and illustrated in Figure 1, the methods used for determining the learning of self-regulation with NF-training are heterogeneous. By “learning” (or “EEG-learning”) we will refer to an improvement in a targeted electrophysiological parameter measuring self-regulated brain activity across time, while “EEG training response” implies more generally any training-related change of an electrophysiological parameter (see Section Baseline increments). We will present a brief overview over different methods and learning indices used in the reviewed studies, discuss possible problems and present additional approaches from studies with other groups than ADHD.

### Units of Measurement

The most commonly used units of measurement are the mean level of *amplitude* and the *percentage of time* beyond a predefined threshold of EEG activity. The amount of decrease or increase of amplitude in the desired direction or the increased amount of time spent in the desired range of frequencies should reflect the participant’s improved regulation efficiency across time. Often, regulation success is dichotomized (yes or no) on each trial, and hit rates are computed online and presented as reinforcers (bonus points) after a block of trials. Such hit rates may be used to represent the EEG learning success across time (e.g., hits above threshold per minute, for children with high functioning autism see Pineda et al., 2014). This requires, however, that criteria for hits/reward are kept stable, which is not the case with adaptive programs or shaping. Moreover, the use of time units above threshold as criterion is not sensitive to smaller improvements in the regulation of amplitudes just below the threshold.

When considering SCP-NF, the observation of only the change in mean amplitude provides no direct evidence about the participant’s ability to differentiate between a state of activation (reflected by a negative amplitude) or deactivation (reflected by a positive amplitude). Nevertheless this skill is hypothesized to be the main training goal in SCP-NF. For the evaluation of progress in learning to differentiate between polarities, it has been common to compute the difference between the means of positive or negative amplitudes and then compare these across sessions (Strehl et al., 2006; Drechsler et al., 2007; Doehnert et al., 2008). However, this method alone fails to account for cases in which regulation has only been achieved in one direction. To illustrate this, it might be the case that the participant mistakenly produces an amplitude of moderate negative polarity during the positivity trial, while the performance in the negativity trial is correct (i.e., strong negative polarity) (see Blume, 2012). This objection especially accounts for ADHD-patients, as in several studies cross-sessional learning has been reported only for negativity, but not for positivity trials (Strehl et al., 2006; Drechsler et al., 2007; Leins et al., 2007; Gani et al., 2008).

### Cross-Session Learning

In the ADHD studies reviewed, the calculation of cross-session learning was based on different samplings of time periods: Several studies used two time periods (session 1 and session 40; Russell-Chapin et al., 2013) or a small number of selected sessions, usually consisting of one from the beginning, one or two in the middle and one from the end of the training course (1st, 5th, 9th, 13th session: Gevensleben et al., 2014; 1st, 10th, 20th, 30th session: Vollebregt et al., 2014; Figure 1A). However, sampling only a small number of single sessions for the calculation of learning is often problematic as the performance of a single session may be biased due to external variables unrelated to the training (i.e., motivation in the final sessions might be lower, day-to-day events, sleep patterns, etc.). In addition, several studies reported large variability in intra-individual learning performance (Strehl et al., 2006; Drechsler et al., 2007; Leins et al., 2007; for healthy participants e.g., Gruzelier et al., 2014a; Wan et al., 2014). To reduce this large variability throughout the course of the training, some researchers clustered groups of sessions into blocks for analysis, e.g., two sessions into one block (sessions 2/3, sessions 29/30 and follow-up: Strehl et al., 2006; Leins et al., 2007; Gani et al., 2008) or all sessions into three blocks of 10 sessions (Bakhshayesh et al., 2011). Alternately, only the second half of the sessions was incorporated into the (sub-) analysis, as this later phase was thought to be more indicative of learning progress than the first half (Drechsler et al., 2007; epileptic patients: Kotchoubey et al., 1999).

In other studies, training performance has been considered across all sessions, which allows for a more fine-grained analysis of the course of learning also including non-linear changes (Figure 1B; ADHD patients: Lubar et al., 1995; Hillard et al., 2013; for NF learning curves in studies with other clinical groups see e.g., Kouijzer et al., 2013; Enriquez-Geppert et al., 2014; Pineda et al., 2014; Wan et al., 2014). Strehl et al. (2005) argue that a steady learning curve across sessions is not necessary to qualify as a learner, as some subjects might find an optimal strategy only at the end of training.

Large intra-individual variability in cross-sessional EEG regulation performance has also been reported in studies with healthy adults and has been attributed to fluctuating arousal levels. Gruzelier et al. (2014a) refer to healthy participants’ self-reported irregularities in night sleep. Indeed, there is evidence that ADHD patients in particular suffer from sleep irregularities (Spruyt and Gozal, 2011). However, the variability of performance due to fluctuations in motivation and arousal is a major feature of ADHD. In order to account for the intra-individual variability of learning performance, Strehl et al. (2006) normalized the data by dividing the individual mean NF-parameters by the individual standard error. This procedure reduces the likelihood of a bias towards subjects with high amplitudes in group analyses of learning. To illustrate this bias, one can imagine a subject with a slow gradual increase in amplitude and thus a small standard deviation. Without normalization, this subject is less likely to reach a predefined criterion of good learning than another subject with a fluctuating pattern.

### Within-Session Learning

Both within- and cross-session EEG-learning (decrease in theta/low beta and theta/alpha ratios) was reported in ADHD-patients by Hillard et al. (2013), using a wide band EEG regulation training at a prefrontal site. Within-session analyses for theta/low beta ration and theta/alpha ratio resulted in significant decrease in the shape of a logarithmic curve over the 25 min of training (for illustration see Figure 1G). In addition, significant progressive changes in the expected direction across sessions were found for all analyzed frequencies. Bink et al. (2014) found larger within-session decrease of theta activity during the last sessions of a NF-FR theta/SMR training compared to the first ones, but no significant change of mean power across sessions. Escolano et al. (2014) analyzed within-session learning in an individualized upper alpha training for children with ADHD. Before and after each session QEEGs were recorded with eyes closed (resting EEG, passive baseline) and with eyes open while performing a visual counting task (active baseline). An unexpected pre-post session decrease was found for counting task related EEG activity (alpha “rebound” effect), in contrast to findings by the authors with healthy adults (Escolano et al., 2011).

Different approaches exist to measure within-session learning, e.g., by relating the mean NF-parameters of each period within a session to the first (Wan et al., 2014) or preceding period (Egner and Gruzelier, 2001), collapsed across sessions. Alternatively, a period or a complete session may be divided into very short segments and collapses across sessions (Dempster and Vernon, 2009) or the change of within-session mean parameters may be analyzed across sessions (Cho et al., 2008; Figure 1F). Although it might initially seem counterintuitive to examine within-session learning regarding long-term outcome and specificity, there is evidence from NF-studies with healthy individuals that within-session learning collapsed across sessions may be correlated with outcome (Ros et al., 2009). Gruzelier (2014b) argues that the consideration of within-session learning would result in a more robust measure of learning than cross-session learning alone, because the overall error variance might be smoothed by a smaller sampling rate of the data within one session averaged over multiple sessions. Several studies with healthy individuals which included both within- and cross-session learning either failed to show cross-sessional NF-learning at all (Hardman et al., 1997; Cho et al., 2008) or only found a trend (Gruzelier et al., 2014b). By contrast, within-session learning was often evident, i.e., participants improved throughout the session. These findings suggest that it might be interesting to include within-session analyses—or cross-session changes of within-session learning, respectively—more systematically in future NF studies with ADHD.

### Baseline Increments

There is increasing evidence from NF studies with healthy adults, that NF may have a strong impact on baseline QEEG, sometimes stronger than on the targeted electrophysiological parameter fed back during the training (Hanslmayr et al., 2005; Ros et al., 2009). As a consequence, EEG-learning should be reflected by a change in pre-session EEG baselines throughout the training course (Gruzelier, 2014b). However, only very few NF-studies with ADHD children examined pre-session or pre-post-session changes in EEG spectra. Bakhshayesh et al. (2011) compared session baselines of the first, second and third section of the training and found larger effects for baseline than for feedback parameters. Maurizio et al. (2014, see also Liechti et al., 2013) reported that after combined NF-SCP and NF-FR with tomographic EEG, individual pre-session baseline values gradually converged towards the group mean across sessions, which was interpreted as normalization (Figure 1E). In an individualized upper alpha-NF for children with ADHD, Escolano et al. (2014) recorded pre- and post-session QEEG and found a significant increase in power across sessions in the targeted parameter in an active pre-session QEEG condition, i.e., when children performed a counting task, while no significant increase in alpha power was obtained either during training or pre-session eyes closed resting EEG.

Several other NF-alpha studies with healthy subjects have shown that by recording a resting-baseline both before and after the training session, the incremental curves constructed from these data provided a more complete picture of the EEG training response over time (Figure 1D; Cho et al., 2008; Escolano et al., 2011; Zoefel et al., 2011; Kouijzer et al., 2013). First, within a training session, the post-session baseline was usually larger than the pre-session baseline. This could be interpreted as a measure of improvement within the session. Second, the overall learning progress achieved during one session was built upon the progress achieved in the previous session. In other words, the baseline measured at the beginning of a session was on the same level as the post-sessional baseline of the previous session. This ratchet-like linear increase in resting baseline seems to indicate that regulation skills are improving throughout the course of the training (Escolano et al., 2011; Figure 1D). A possible consequence from this finding is that EEG learning across sessions may be masked by progressive increments in resting baseline if these increments are not taken into account in the analysis of change. Compared to the training performance at the first session, target amplitudes may show a cross-sessional increase, even when no increase can be found when considering within-sessional mean amplitudes relative to their respective pre-session baselines (Figure 1C). Although this remains to be demonstrated for NF with ADHD, NF-alpha-studies with healthy adults lend support to this hypothesis (Dempster and Vernon, 2009). Incorporating a baseline measure might also enhance the comparability of learning performance on group level. For instance, in a NF-study with insomnia patients, (Schabus et al., 2014; also see Hoedlmoser et al., 2008) divided the session mean amplitude of a subject by the corresponding pre-session baseline. As a result, transforming the data into a relative instead of an absolute value may smooth out the high inter-subject variability of baseline measures.

### Classification of Good and Poor Learning

Whereas most of the reviewed ADHD-studies analyse learning improvements of EEG regulation with regard to the full treatment group (Bakhshayesh et al., 2011; Russell-Chapin et al., 2013; Escolano et al., 2014; Gevensleben et al., 2014) some studies report the rate of learners (or responder rate) (Lubar et al., 1995; Kropotov et al., 2005; DeBeus and Kaiser, 2011), or distinguish between good and poor performers (Drechsler et al., 2007) (or successful and unsuccessful regulators; Strehl et al., 2006), in order to analyse learning outcome. However, in several NF-ADHD studies which do not include the analysis of EEG learning, the term “responder rate” is used with regard to the clinical outcome, which is usually defined by the reduction in ADHD symptoms (e.g., Gevensleben et al., 2009).

In studies which report the rate of learners, training success may be defined by a fixed criterion, e.g., a *percentage cut-off* in order to classify participants as learners if they have reached a predefined criterion in a fixed percentage of sessions. These cut-off values for successful learning often appear to be chosen *ad hoc* (e.g., Kropotov et al., 2005), or may be taken from previous studies (e.g., Weber et al., 2011, for NF with healthy adults). In a theta/beta training, Kropotov et al. (2005) defined successful learning by an increase in amplitudes of at least 25% during feedback periods compared to resting periods in at least 60% of all sessions. This definition resulted in 82% participants being classified as “good performers”. The number of training sessions for each patient varied from 15 to 22, depending on several factors such as age, type of ADHD, learning curves, and parent reports. The termination criteria were (1) stabilization of training performance assessed by the dynamics of the trained parameter during the last three to five sessions; and (2) stabilization of patient’s behavior according to parent reports. Lubar et al. (1995) and DeBeus and Kaiser (2011) used a relative change of NF-parameters as a criterion for categorizing performance. In this approach, subjects are classified as good performers when performance in the final training sessions is significantly superior to that in the first ones or when NF parameters increased across all sessions. Lubar et al. (1995) reported a responder rate of 65% in theta/beta NF-FR training, defined by significant negative correlation of theta by session number. DeBeus and Kaiser (2011) found 74% of responders in NF-FR training, defined as an increase of half a standard deviation in the Engagement Index (beta/theta + alpha) from session 1–3 to 18–20. (For studies with healthy participants see Vernon et al., 2003; Weber et al., 2011; Zoefel et al., 2011; Dekker et al., 2014).

A different approach is to employ a cut-off value defined by the median split of the learning parameter (Strehl et al., 2006; Drechsler et al., 2007; Doehnert et al., 2008) which allocates the participants into a group of good and a group of poor learners. Naturally, in this case no meaningful responder rate can be given. Moreover, learners and non-learners do not have to be equally distributed, contrary to what the use of median split may lead one to presume. As a consequence, the variability of learning performances may vary considerably in both groups. Evidently, given these methodological differences in the calculation of good learning in the aforementioned studies, it is difficult to draw meaningful conclusions about the average responder rate in ADHD NF. According to a study by Monastra et al. (2002), EEG learning essentially appears to be a matter of time. Only children with predefined QEEG abnormalities were included in their study and treatment was continued until the criterion for EEG learning had been obtained in each individual case (“normalization”, i.e., a degree of cortical slowing within 1.0 SD of age peers). Therefore all participants reached the criterion, which is equivalent to a responder rate of 100%, but the number of sessions varied considerably among the participants. Further evidence that time may matter with regard to the classification of good and poor learning of EEG self-regulation comes from studies indicating that regulation skills might continue to develop and consolidate long after the end of the training (Blume, 2012; for NF with epilepsy see Strehl et al., 2005).

## Failing to Learn

Some studies on NF in ADHD which investigated EEG learning performance failed to find the expected significant changes on group level. In a double blind placebo controlled study using Q-EEG feedback with individualized protocols, Vollebregt et al. (2014) compared mean power of the trained frequency bands of the first, tenth, twentieth and final session. The authors report that seven out of ten children showed changes in power toward the directed target, but no child showed changes in more than one frequency band, and that all children also presented changes away from a training target in some bands. Clinical responders (defined by behavioral improvements) showed EEG changes in both desired and non-desired directions. In a study using tomographic NF, including both NF-SCP and NF-FR, the authors failed to find significant EEG learning on group level (Liechti et al., 2012; Maurizio et al., 2014). Besides methodological aspects, the fact that the regulation of a brain area which is known to be underactivated in ADHD, the anterior cingulate cortex, was fed back, may have presented a special difficulty for the participants. However, in this study patients displayed individual changes towards normalization of pre-session baselines across sessions (Figure 1E).

Whether or why individual children might fail to learn self-regulation of brain activity has not been the central focus of ADHD-NF research. These questions have been tackled more comprehensively in the Brain Computer Interface (BCI) research, which aims at training individuals to control technical devices via the regulation of brain activity, e.g., to use a communication computer or to navigate a wheelchair controlled by the modulation of brain waves (Guger et al., 2003; Blankertz et al., 2010; Vidaurre and Blankertz, 2010). While neurofeedback is based on operant conditioning with a fixed-target EEG signal, BCI most often uses a machine learning approach. This means that the EEG signal is optimized according to the participant’s brain activity during the task (Lotte et al., 2013). Nevertheless, a substantial portion of participants, 10–30%, fail to gain control, which has been referred to as BCI “illiteracy” (Dickhaus et al., 2009) or “inefficiency” (Kübler and Müller, 2007). Allison and Neuper (2010) presume that a small number of probands may display individual brain structures, which, although not pathological, may not allow the recording of a target EEG parameter by normal surface electrodes (see also Halder et al., 2013). If proper calibration does not help in adapting to individual morphology, the solution is to switch to a different EEG parameter or neuroimaging technology. It is possible, however, that the patient will not be able to use BCI at all. Otherwise, one should try to improve the accuracy of the BCI procedure, e.g., by improving the selection of the existing brain signals through approved algorithms or by incorporating better error correction (Allison and Neuper, 2010). The authors hypothesize that BCI illiteracy might be confined to certain techniques or tasks in a particular individual while the same person may possibly perform better in another paradigm. All of these points are concerned with methodological and technical aspects, while, as the authors state, variables such as mood, motivation, distraction, and test setting may also play a role. In patients with Amyotrophic Lateral Sclerosis (ALS), motivational factors such as challenge and mastery confidence were positively correlated with BCI performance (Nijboer et al., 2008). However, an exaggerated feeling of self-efficacy may constitute an impediment rather than a help for good NF performance. Witte et al. (2013) reported that SMR-learning performance was negatively correlated with the attribution of locus of control. Participants whose confidence in control over a technical device was low performed better than those with a high belief of control. This effect was explained by a possible cognitive overload when controlling a technological device, which in turn might adversely affect the relaxation states which SMR-training aims to achieve. In a study on psychological predictors of SMR learning, the best predictor of SMR performance were objective measures for the accuracy of fine motor skills and the ability to concentrate on the task (Hammer et al., 2012), whereas subjective factors, such as well-being, did not predict performance. This was explained by the fact that only healthy individuals, consisting mostly of students, participated in the study.

To which extent these results from BCI research also apply to NF with ADHD and whether a proportion of children might be unable to learn EEG regulation with one protocol but might gain control with another, is unknown. In future studies, more attention should be paid to the question of whether and why children with ADHD might fail to learn self-regulation of brain activity.

## Learning Patterns of Self-Regulated Brain Activity

One crucial question is how to interpret patterns of learning curves in terms of learning performance, and whether it is possible to distinguish characteristic learning patterns in ADHD. For the time being, the extent to which the learning of EEG regulation in ADHD may be expected to be progressive and regular remains unclear. Differences in the training administration of ADHD-NF studies (session frequency, time intervals between sessions, number or duration of trials per session, training breaks etc.) and the small number of patients in many studies make it difficult to draw conclusions. For theta/beta-NF, Lubar et al. (1995) (40 sessions) as well as Bakhshayesh et al. (2011) (30 sessions) observed an increment in performance during the first training phase, followed by a stagnation phase in the middle of the training and a subsequent increase in performance in the final third of training sessions. In an SCP-training (Blume, 2012; 25 sessions; 4 weeks-break between session 12 and 13), children with ADHD displayed a stagnation in the second compared to the first training phase, while performance was enhanced again at the 6-months follow-up. Interestingly, some of the children who had been classified as non-learners after the second training phase, showed good EEG performance at follow-up (see Strehl, 2014). These learning patterns—stagnation and subsequent increased performance after a break or in the final part of the training—have been discussed in terms of the individual speed of learning and a related overtraining-effect which might occur earlier for fast learners than for slow learners (Blume, 2012). In several studies with healthy participants, NF-FR learning has been reported to reach a plateau after 4–6 sessions with a subsequent stagnation (total session number 8–10) (Ros et al., 2009; Gruzelier et al., 2010; Keizer et al., 2010; Dekker et al., 2014; Enriquez-Geppert et al., 2014). These plateaus have been hypothesized to reflect training fatigue or over-learning. Patients’ learning curve patterns might differ from those of healthy subjects. For instance, Kübler et al. (2004) found that healthy subjects reached a learning plateau after 3 sessions, whereas in patients with ALS, no learning plateau was reached after 12 sessions. In an NF-study with primary insomnia patients, participants displayed fluctuating learning, which, intercepted by sessions of stagnation, increased across sessions (Schabus et al., 2014). In anxiety patients, Hardt and Kamiya (1978) postulated a fifth-order learning curve, starting with an initial increase, and followed by a dip, a second increase, and a final exponential increase for alpha-NF learning.

In healthy individuals, learning curve patterns have been shown to distinguish non-learners from good learners, showing not only a plateau, but also a decrease of performance: Poor SMR performance was associated with a highly significant 10% decrease in NF-parameters during the second training phase when compared to the first (Ros et al., 2009). A further finding of this study was that smaller intervals between sessions seemed to lead to better EEG learning than longer intervals, indicating that an intense training rhythm may be advantageous.

It should be kept in mind that learning patterns in ADHD besides being extremely individual in nature, may also substantially depend on factors of the setting, such as the relation to the therapist, motivation, external support (Monastra et al., 2002; Drechsler et al., 2007; Strehl, 2014). For the time being, there is a lack of studies that describe characteristic learning patterns and possible subgroups of learners in ADHD which would allow to select the training protocol or to systematically adapt the program according to the learning type of the child.

## The Association between Self-Regulated Brain Activity and Clinical Outcome Gains

The few studies that examined the association between NF-learning and the clinical outcome in ADHD (see Table 1) used heterogeneous methods. Participants may be categorized in poor and good learners for subsequent data analysis or classified according to good and poor clinical outcome, while in other studies no such distinctions are drawn.

For instance, Strehl et al. (2006) defined criteria for good SCP-learning (negativity learning, calculated by median split) as well as for good clinical outcome in ADHD (at least a 2-point reduction in either hyperactivity or inattention according to DSM-IV) and reported a statistically significant association between the two measures at the end of the training. At the 6-months follow-up, the association between clinical outcome and NF-learning still almost reached significance, indicating a long lasting effect of the training. Drechsler et al. (2007) reported a positive correlation between the pre-post decrease in parent-rated ADHD symptoms and the ability to differentiate between SCP positivity and negativity trials. This association was confined to the group of good performers, defined by median split, whereas in poor learners, ADHD symptomatic improvements were uncorrelated with SCP performance. In NF-FR training, DeBeus and Kaiser (2011) reported a significant correlation between improved EEG regulation and teacher ratings of ADHD symptoms, which was also confined to the group of good performers. Recently, Gevensleben et al. (2014) conducted an SCP-NF study with ADHD children, and found a correlation between the pre-post change in parent-rated inattention symptoms and the increase in negativity from the first to the fifth session and from the first to the ninth session. This study was based on a small sample (*n* = 10) and the authors did not distinguish between good and poor performers.

Several studies have analyzed the association between EEG learning and neuropsychological outcome. Kropotov et al. (2005) reported that learning to enhance beta and SMR in ADHD correlated with a significant decrease in response time and variability of response time in a Go/No-Go task only for good performers. Lubar et al. (1995) reported stronger improvements on a computerized attention test for learners than for non-learners after NF-FR training.

The relationship between positive clinical outcome and successful NF learning has been confirmed in a number of NF studies with other clinical groups, such as patients with epilepsy (Daum et al., 1993; Kotchoubey et al., 1997; Strehl et al., 2005) or sleep disorder (Schabus et al., 2014). In healthy subjects, NF-learning correlated positively with improvement in short-term memory (Nan et al., 2012), mental rotation (Hanslmayr et al., 2005), microsurgical skills (Ros et al., 2009) and enhancement in cognitive creativity (Gruzelier, 2014a).

However it should be kept in mind, that the relationship between successful regulation of an individual’s brain activity and positive clinical outcome is not reciprocal: Improvements in parent-rated ADHD symptoms are not confined to learners (Drechsler et al., 2007), indicating that non-specific treatment effects also contribute to the clinical outcome.

## Electrophysiological Pre-Post Changes, Protocol Specific Effects and Prediction

In NF research with ADHD patients, to date no study has directly related pre-post electrophysiological changes to increments in NF performance across sessions.

However, several studies have reported pre-post effects on electrophysiological levels, although most of them did not analyse EEG learning across sessions. Often, these studies focus in a hypothesis-driven manner on electrophysiological measures related to the feedback protocol used, examining pre-post Q-EEG changes after NF-FR with special emphasis on the trained frequency (e.g., Thompson and Thompson, 1998; Pop-Jordanova et al., 2005) and pre-post contingent negative variation CNV or other ERPs after NF-SCP (e.g., Heinrich et al., 2004; Mayer et al., 2012). There is evidence that training protocols may result in specific effects which, at least indirectly, supports the importance of successful and differential learning of EEG regulation with regard to pre-post EEG changes. Wangler et al. (2011) and Gevensleben et al. (2009) compared NF-SCP and FR-NF training in a crossover design and examined electrophysiological effects of both protocols. They reported pre-post increase in the CNV after NF-SCP but not after NF-FR. According to pre-post QEEG analyses, both protocols resulted in a decrease in theta bands activity. Despite this evidence of protocol-specific effects on EEG, it might be advisable to explore the full frequency spectrum or to include additional measures in the pre-post EEG analyses. Several studies, mostly with healthy participants, demonstrate that electrophysiological pre-post effects are not necessarily confined to the targeted training parameter (for a detailed review, see Gruzelier, 2014b). An example with ADHD patients is provided by Doehnert et al. (2008) who conducted SCP training and reported a pre/post QEEG theta decrease at Oz, while they did not find the expected effects on the CNV. Another evidence for extended effects comes from a study by Escolano et al. (2014) who in an alpha-NF analyzed the course of pre- and post-session QEEG in resting and in task-related states, though with a focus on the target frequencies. Cross sessional changes in the expected direction were limited to task-related pre-session QEEG while changes in pre-session resting EEG were not significant. Liechti et al. (2012) were unable to find any significant association between changes in ADHD symptoms and cross-session NF-learning. However, they reported specific associations between cross-session changes in baseline-frequencies and outcome gains, such as a positive correlation between theta/beta increases in specific regions and frontal beta decreases with reductions in hyperactivity/impulsivity. The extent to which in the case of generalized and extended EEG training response the electrophysiological outcome should still be considered the result of a specific training effect should be the subject of a more refined methodological debate.

Electrophysiological pre-post changes have been related to clinical outcome, which indicates that electrophysiological change is reflected by behavioral improvement (Doehnert et al., 2008; Gevensleben et al., 2009; Wangler et al., 2011; Arns et al., 2012). Still, electrophysiological pre-post measures do not directly reflect EEG regulation performance during feedback trials. Pre-post changes in electrophysiological markers have also been reported after mindfulness training (Moore et al., 2012; Schoenberg et al., 2014), which shares several therapeutic characteristics with the NF setting, and thus results based on these measures do not provide the best indication of NF specificity.

Studies that analyze initial EEG learning patterns across or within sessions with regard to overall EEG learning performance, are rare. However, the identification of early predictors of Nf learning would be very helpful in terms of providing a better basis for therapeutic decision-making or adapting the training protocol accordingly. In an unpublished doctoral thesis by Goth (2006) on NF training in children with ADHD, the mean amplitudes of negativity trials in session 1 and 2 were the best predictors of subsequent improvements in SCP-NF-regulation performance, whereas a large number of inattention symptoms predicted poor EEG learning. In NF-FR training, a similar trend was found for successful regulation in early sessions. The best predictor of EEG learning success in NF-FR, however, was a high IQ.

In patients with ALS, good performance at an early training stage of SCP regulation was correlated with subsequent good learning (Neumann and Birbaumer, 2003). In a study with healthy adults, it could be shown that certain morphological parameters may have a beneficial effect on training success: Frontal-midline theta NF-learning was predicted by the volume of the mid-cingulate cortex and the white matter concentration of underlying brain structures (Enriquez-Geppert et al., 2013).

## Is it Possible to Promote EEG Self-Regulation Performance?

It has been suggested that children with ADHD might require explicit rather than implicit learning (Lansbergen et al., 2011). According to several authors in the field, children with ADHD need to actively practice mental strategies to self-regulate brain activity and have to be instructed on how to translate the newly learned skill into everyday life (Gevensleben et al., 2009; Heinrich and Gevensleben, 2013; see Strehl, 2014). They suggest that during the first lessons of training, the trainer should encourage the child to find an appropriate strategy (“I imagine I’m waiting for the starting signal in a race”). This initial strategy should be gradually reduced and finally abandoned in the course of the training, when regulation becomes automatized (Heinrich and Gevensleben, 2013). To the best of our knowledge the impact of instruction and explicit strategy training on EEG training performance has not been systematically investigated in ADHD. Gevensleben et al. (2014) hypothesize that the use of different transfer instructions for children with Tic disorder than for children with ADHD may have resulted in specific clinical outcome gains in inhibitory control. However, these setting differences did not apply to the self—regulation during feedback trials, but to the transfer outside the laboratory. Whether self-regulation of brain activity may be helped or exacerbated by the use of conscious top-down strategies is unclear and probably also depends on specific protocols. As SCP training aims at quick changes in polarity, it may be expected that top-down regulation plays a more prominent role here than in NF-FR (see Loo and Makeig, 2012). Arguments both for and against the promotion of conscious strategy use and the importance of self-awareness for NF performance come from research with healthy subjects and other clinical groups. Neurofeedback has been hypothesized by several researchers to involve an increased awareness of the physiological states underlying the feedback (Plotkin, 1981; Congedo, 2007). Recent evidence for this hypothesis is provided by a study on EEG discrimination training with healthy adults (Frederick, 2012). After a baseline recording (150 s), subjects had to respond to a prompt asking whether in that moment they were in a low (<30th percentile of the baseline) or high alpha state (>70th percentile). They immediately received feedback about their guess. 75% of participants showed a significant learning curve and were successful in discriminating their brain activity states. There might be a reciprocal relationship between discrimination of brain states and the training of brain state regulation, as Cinciripini (1984) showed for SMR and Kotchoubey et al. (2002) demonstrated for SCP-training. Moreover, successful regulation skills might also have a positive impact on the discrimination ability of brain regulation states. Gruzelier (2014a) reports that the subjects’ first positive self-judgment about their ability to regulate SMR ratios occurred close to the time, when their learning curve reached a plateau.

A further question concerns whether and how mental strategies might affect NF-learning. Nan et al. (2012) reported that their (healthy adult) alpha-NF subjects favored positive mental strategies (e.g., friends, love, family) which they estimated the most successful. However, these subjective judgments were not related to the actual NF-performance. The effects of strategy use might also depend on the frequency band: in NF-SMR training with healthy adults, participants who used no mental strategy at the end of the training performed better than those who did, thus indicating a possibly counterproductive effect of strategy use on SMR learning. In contrast, strategy use had no influence on gamma learning (Kober et al., 2013). Neumann and Birbaumer (2003) argue that providing patients with initial strategies may promote self-regulation at the beginning of training but would prevent subjects from trying out other potentially more effective strategies with training progress. This argument is in line with Witte et al. (2013) who emphasize the importance of the initial trial-and-error learning, which due to “immediate closed-loop feedback” could ameliorate the subjects’ regulation skills. This unconscious adapting to the desired state might thereby become automated.

To conclude, the literature provides arguments both against and in favor of a more systematic approach to foster EEG learning and self-awareness of EEG activity states in children with ADHD. It might be worthwhile to devote more attention to the question of whether and how the learning of EEG self-regulation can be systematically promoted in children with ADHD.

## Conclusion

Discussions about NF specificity need to include analyses of EEG regulation performance and its impact on clinical outcome. Besides its effects on ADHD primary symptoms, associations with factors usually regarded as “generic effects”, such as improved self-perception or self-efficacy should also be considered. To provide optimal conditions for learning, it is necessary to improve our knowledge regarding characteristic cross-session learning trajectories and within-session performance in ADHD and to adapt training schedules accordingly. This also includes possible therapeutic strategies which might promote EEG self-regulation in children with ADHD. In the future, NF devices used for NF research with ADHD should adhere to more rigorous scientific standards, allowing for qualitatively acceptable EEG recording during treatment sessions, including artifact control, in order to document learning of EEG self-regulation. From a scientific point of view, the current practice, which allows the use of NF devices of uncertain quality or protocols based on undisclosed algorithms for NF research, is unsatisfactory. It is bewildering that, with regard to the evaluation of efficacy and specificity of NF, strictest methodological standards are demanded for the study design, while no scientific standards need to be applied to the treatment. Several meta-studies (Arns et al., 2009; Hodgson et al., 2014) have demonstrated the efficacy of NF with regard to the improvement of ADHD symptoms. Whether NF is efficacious AND specific still needs further investigation, which should go beyond analyzing pre-post changes and include analyses of the treatment process and the learning of EEG self-regulation.

## Conflict of Interest Statement

The authors declare that the research was conducted in the absence of any commercial or financial relationships that could be construed as a potential conflict of interest.
